# MicroRNA-18b inhibits the growth of malignant melanoma via inhibition of HIF-1α-mediated glycolysis

**DOI:** 10.3892/or.2021.8161

**Published:** 2021-07-30

**Authors:** Yao Chen, Ziqing Zhang, Chengqun Luo, Zizi Chen, Jianda Zhou

Oncol Rep 36: 471-479, 2016; DOI: 10.3892/or.2016.4824

Following the publication of this paper, it was drawn to the Editors attention by a concerned reader that certain of the flow cytometric data shown in Fig. 3, and western blotting assay data shown in Fig. 6F, were strikingly similar to data appearing in different form in other articles by different authors. Owing to the fact that the contentious data in the above article had already been published elsewhere, or were already under consideration for publication, prior to its submission to *Oncology Reports*, the Editor has decided that this paper should be retracted from the Journal. The authors were asked for an explanation to account for these concerns, but the Editorial Office did not receive any reply. The Editor apologizes to the readership for any inconvenience caused.

